# Integrating High-Resolution Mass Spectral Data, Bioassays and Computational Models to Annotate Bioactives in Botanical Extracts: Case Study Analysis of *C. asiatica* Extract Associates Dicaffeoylquinic Acids with Protection against Amyloid-β Toxicity

**DOI:** 10.3390/molecules29040838

**Published:** 2024-02-13

**Authors:** Armando Alcázar Magaña, Ashish Vaswani, Kevin S. Brown, Yuan Jiang, Md Nure Alam, Maya Caruso, Parnian Lak, Paul Cheong, Nora E. Gray, Joseph F. Quinn, Amala Soumyanath, Jan F. Stevens, Claudia S. Maier

**Affiliations:** 1 Department of Chemistry, Oregon State University, Corvallis, OR 97331, USA; armando.alcazarmagana@ubc.ca (A.A.M.); ashishvaswani677@gmail.com (A.V.); alammdn@oregonstate.edu (M.N.A.); parnian.lak@gmail.com (P.L.); cheongh@oregonstate.edu (P.C.); 2 BENFRA Botanical Dietary Supplements Research Center, Oregon Health & Science University, Portland, OR 97239, USA; grayn@ohsu.edu (N.E.G.); soumyana@ohsu.edu (A.S.); fred.stevens@oregonstate.edu (J.F.S.); 3 Life Sciences Institute, University of British Columbia, Vancouver, BC V6T 1Z4, Canada; 4 Department of Pharmaceutical Sciences, Oregon State University, Corvallis, OR 97331, USA; kevin.brown@oregonstate.edu; 5 School of Chemical, Biological, and Environmental Engineering, Oregon State University, 116 Johnson Hall, 105 SW 26th Street, Corvallis, OR 97331, USA; 6 Department of Statistics, Oregon State University, Corvallis, OR 97331, USA; yuan.jiang@oregonstate.edu; 7 Department of Neurology, Oregon Health & Science University, Portland, OR 97239, USA; maya.caruso1@gmail.com (M.C.); quinnj@ohsu.edu (J.F.Q.); 8 Parkinson’s Disease Research Education and Clinical Care Center, Veterans’ Administration Portland Health Care System, Portland, OR 97239, USA; 9 Linus Pauling Institute, Oregon State University, Corvallis, OR 97331, USA

**Keywords:** *Centella asiatica*, bioactives, bioassays, neuroprotection, computational methods, Elastic Net, spectral networks, GNPS

## Abstract

Rapid screening of botanical extracts for the discovery of bioactive natural products was performed using a fractionation approach in conjunction with flow-injection high-resolution mass spectrometry for obtaining chemical fingerprints of each fraction, enabling the correlation of the relative abundance of molecular features (representing individual phytochemicals) with the read-outs of bioassays. We applied this strategy for discovering and identifying constituents of *Centella asiatica* (*C. asiatica*) that protect against Aβ cytotoxicity in vitro. *C. asiatica* has been associated with improving mental health and cognitive function, with potential use in Alzheimer’s disease. Human neuroblastoma MC65 cells were exposed to subfractions of an aqueous extract of *C. asiatica* to evaluate the protective benefit derived from these subfractions against amyloid β-cytotoxicity. The % viability score of the cells exposed to each subfraction was used in conjunction with the intensity of the molecular features in two computational models, namely Elastic Net and selectivity ratio, to determine the relationship of the peak intensity of molecular features with % viability. Finally, the correlation of mass spectral features with MC65 protection and their abundance in different sub-fractions were visualized using GNPS molecular networking. Both computational methods unequivocally identified dicaffeoylquinic acids as providing strong protection against Aβ-toxicity in MC65 cells, in agreement with the protective effects observed for these compounds in previous preclinical model studies.

## 1. Introduction

Plants produce more than 200,000 distinct specialized metabolites [1], constituting the principal reservoir of bioactive compounds combating diseases in numerous countries [2,3]. Nevertheless, the complexity of working with plant extracts lies in discriminating between the specialized metabolites responsible for producing biological activity in bioassays, preclinical models, or humans. The traditional bioassay-guided fractionation approach is tedious and time-consuming [4,5]. This approach requires the separation of certain phytochemicals based on physicochemical properties such as polarity, charge, or size, and assessing the bioactivity in a step-by-step methodology. Carrying out purification and assay stages sequentially may ultimately result in the isolation of a bioactive compound, only to find that the compound has been previously discovered [4,5,6,7,8]. Additionally, there are risks associated with traditional exhaustive fractionation that compounds degrade or become lost during the process [9]. There is an urgent need to accelerate the discovery of natural bioactive products and to remove the dereplication bottleneck. Methods capable of addressing this need are emerging. The Global Natural Product Social Molecular Networking (GNPS) platform can assist in the dereplication and annotation of specialized metabolites [10,11]. Statistical models, such as Partial Least Squares (PLS) models, utilize spectral information to predict bioactive metabolites in complex natural product mixtures [12]. The selectivity ratio method is another well-established tool for assisting in the discovery of biomarkers that utilizes chromatographic and mass spectral profiles [13,14,15]. The selectivity ratio method has been recently applied to the discovery of bioactive constituents in botanical extracts. Recently, our research group showed that Elastic Net (EN), a regularized regression model [16], was capable of correctly predicting the anti-inflammatory bioactive constituents in hop extracts utilizing high-resolution mass spectrometry *m/z* profiles of extract fractions [17].

The objective of the present study was to find and annotate the bioactives in *C. asiatica* extracts that ameliorate cytotoxicity caused by amyloid β in MC65 cells, a cell culture model amenable to high-throughput screening. Aqueous extracts of *C. asiatica* are recognized to enhance memory and mental health [18,19,20,21]. The use of *C. asiatica* preparations in complementary medicine has been associated with ameliorating cognitive decline due to ageing and Alzheimer’s disease [20,22,23,24]. For this purpose, we utilized the Elastic Net method to correlate molecular features (unknown phytochemicals) derived from high-resolution mass spectrometry with the bioactivity levels observed for the *C. asiatica* fraction in the MC65 assay. We selected Elastic Net as it allows for an accurate computation of the contribution of each bioactive phytochemical towards the total bioactivity of the fraction without limiting the number of phytochemicals being used for the prediction [25]. Importantly, we validated the output of the Elastic Net method using the well-established selectivity ratio method. We created a GNPS network that visualizes the association between fraction, bioactivity, and mass spectral data. This case study revealed that mono-and dicaffeoylquinic acids (CQAs) are associated with protecting against amyloid-β toxicity in an MC65 cell model.

## 2. Results and Discussion

### 2.1. Chemical Diversity and Viability in C. asiatica Fractions

The plant-based bioactive compound discovery field is challenged by the need to purify and identify specialized metabolites that exhibit bioactivity in various assays. To address these challenges, our research group developed an innovative approach combining fractionation, high-resolution mass spectrometry, and advanced computational models to rapidly screen plant extracts [17]. An adaptation of this workflow was applied to *C. asiatica* extracts. A critical aspect of our approach involved simplifying the chemical diversity of the plant extract by creating a set of 21 impure fractions, producing distinct compound concentrations across them (fractions A1–A21, Figure S1). The initial liquid–liquid extractions served as a pivotal step in isolating different components of the *Centella asiatica* water (CAW) extract based on their solubility in various solvents. The sub-fractionation approach uses LH-20 chromatography, which allows the separation of phytochemical constituents into low numbers of fractions, thus allowing mass spectral analysis of the chemical constituents while minimizing the matrix effects when analyzing complex mixtures.

**Figure 1:**
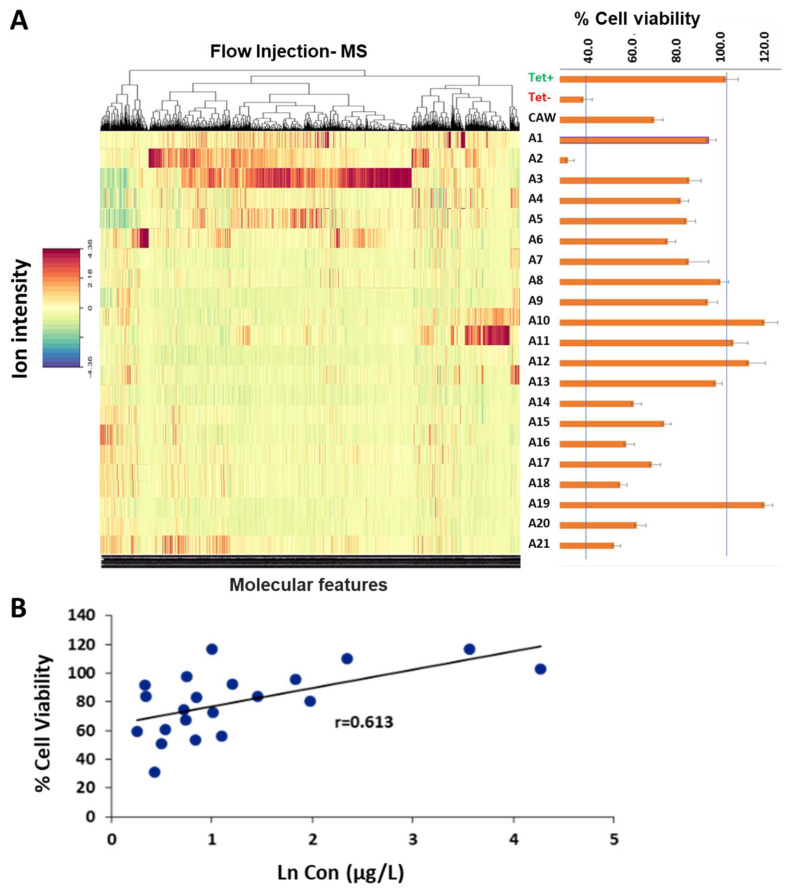
Creating phytochemical variation to detect correlations of individual phytochemicals with biological activity: (**A**) Left—Flow injection–HRMS negative ion mode analysis of 21 CA subfractions. After data processing, over 1500 molecular features (depicted in the heatmap) were aligned according to their molecular masses. Right—% cell viability as an index of protection against Aβ toxicity. Bars represent % viability ± standard error of CAW extract and all subfractions of the CAW extract tested in MC65 cells in the presence of Aβ (induced by absence of tetracycline). (**B**) Correlation of % cell viability with the concentration of di-CQAs (sum of isomers) ([M-H]-, *m/z* 515.12) present in the 21 CA subfractions (each blue dot represents a subfraction). % viability ± standard error assay of CA and all subfraction without tetracycline in MC65 cells (*n* = 12–16).

Mass spectral profiles for the 21 fractions were obtained by flow-injection HRMS (Figure 1). Data were acquired in positive and negative electrospray ionization mode. Over 1500 molecular features were recorded across all fractions, and the gradient of concentrations was computed against the % of proliferation of MC65 cells using two computational models, namely Elastic Net and selectivity ratio (SR). Removing the use of the analytical column in flow injections has the advantage of shortening the analysis time (under 2 min per run) with only 30 s of equilibration time before the next injection. Flow injections increase the potential of the ion suppression phenomenon since all compounds elute together, including sodium and other cations producing several adducts for each compound. However, the sub-fractionation of the plant extracts lessens this problem. Sub-fractionation in conjunction with flow injection has the methodological advantage of minimizing matrix effects due to matrix simplification, allowing the detection of additional molecular features. Nevertheless, careful processing and adduct analysis and deconvolution are needed to obtain mass spectral fingerprints of sufficient quality [26] to feed the computational analysis.

MC65 cells, a neuroblastoma line, express the C-terminal fragment of the amyloid precursor protein (APP CTF) regulated by a tetracycline-responsive promoter. Upon tetracycline withdrawal from the medium, the C99 fragment of APP is expressed, which is then cut by β-secretase to form Aβ peptides. The accumulation of intracellular, endogenous Aβ leads to cell death within 72 h [27]. In the absence of tetracycline, cells treated with each of the 21 fractions exhibit cell viability levels ranging from 5% (A2) to 117% (A10). Remarkably, cells treated with fractions A10, A11, A12, and A19 not only displayed a notable absence of cell death but also exhibited a proliferation surpassing that of the control containing tetracycline, reaching >100% viability. This observation would typically trigger additional analyses to find the compound present in those fractions. However, this is not needed under this methodology. This computational approach has the capability to uncover correlations between variations in the levels of molecular features (phytochemicals) across the fractions and the percentage of viability, thereby identifying the most probable compounds influencing cell viability.

### 2.2. Correlation between Phytochemical Profiles and Neuroprotective Effect

**Figure 2:**
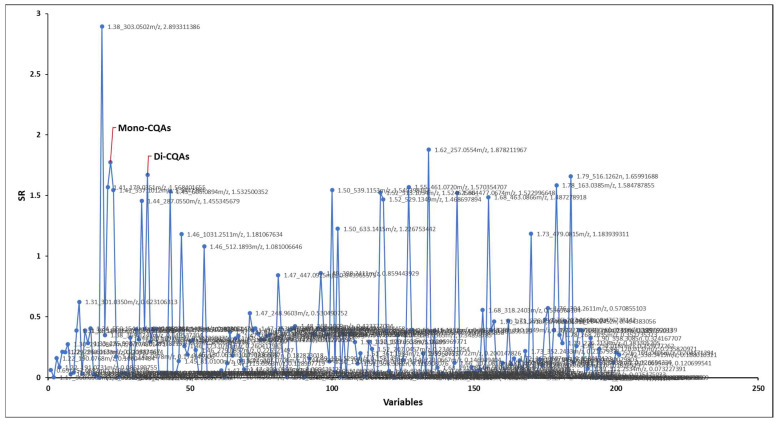
Selectivity ratio analysis. Selectivity ratios of mono- and dicaffeoylquinic acids were 1.66 and 1.78, respectively, positioning them in the top 5 hit list of correlated compounds. The raw SR values can be found in the Supplementary Material (Table S1).

The correlation of the HRMS profiles of the 21 CAW fractions with the neuroprotective activity levels of each fraction was obtained using Elastic Net as previously described [17]. In addition, we applied selectivity ratio analysis to confirm independently the Elastic Net analysis results of putative bioactive compounds acting as inhibitors of Aβ cytotoxicity in the MC65 cell culture model. The selectivity ratio is established by determining the ratio between the explained and residual variances of the spectral variables on the target projected component [13,28]. This ratio serves as a useful tool for selecting variables in the analysis (Figure 2).

The 21 fractions were tested for their protective effect in the MC65 cell culture model of Aβ toxicity, which uses % cell viability as a measure of cell protection. The Elastic Net model identified *m/z* 515.1191 and *m/z* 353.0874 as the top two bioactives, while the selectivity ratio pinpointed *m/z* 303.0502 and *m/z* 257.0554 as the top two bioactive compounds, respectively. The *m/z* values 353.0874 and 515.1191 were previously identified and characterized in *C. asiatica* water extracts as the deprotonated molecular ions (M-H-) of mono- and dicaffeoylquinic acids, respectively [20,29,30]. Despite discrepancies in the top ten bioactive compounds between the two models, six compounds emerged as consistent top candidates in both (Table 1). Notably, the selectivity ratio emphasized the significance of *m/z* 303.0502 [M+H]^+^ and *m/z* 257.0554 as the top bioactives; it also confirmed the identification of *m/z* 515.1191 and *m/z* 353.0874 as the next most important compounds. This validation highlights the effectiveness of Elastic Net in identifying the most likely compounds among the 1500 molecular features.

In predictive modelling for neuroprotective effects, the Elastic Net algorithm presents notable advantages and limitations when compared to other models. Elastic Net’s incorporation of L1 (Lasso regression) and L2 regularization (Ridge regression) facilitates variable selection, making it adept at handling multicollinearity and providing flexibility through parameter tuning. However, Elastic Net’s performance may be compromised when confronted with many irrelevant features and is sensitive to variable scaling (as are many regression models) [17]. On the other hand, the selectivity ratio excels in capturing linear relationships, proving robust against overfitting and capable of handling missing data. The choice between Elastic Net and the selectivity ratio hinges on the specific characteristics of the dataset and the interpretability requirements of the neuroprotective modelling effort. While the *m/z* 515.1191 acids exhibit a high correlation in both models, the other compounds in Table 1 showcase varying degrees of correlation, highlighting the complexity of the botanical extract’s composition. This diversity opens avenues for further exploration in a resource-focused way. In this case study, both systems provide a reduction in the candidates from 1500 molecular features to a handful of them.

Despite the potential limitation of encountering antagonist compounds within the same subfraction, it is noteworthy that this aspect simultaneously highlights a strength of our current strategy, namely the potential of revealing synergies among compounds. This capability to uncover synergistic interactions is critical in investigating bioactive compounds [31]. The intricacies of exploring synergy and antagonism present significant challenges, especially in complex natural product chemistry [32]. The conventional approach in this field focuses on simplifying complexity and isolating single active constituents for drug development, thereby making the comprehensive study of synergistic and antagonistic interactions notably challenging.

Finally, quite often, one of the struggles of using traditional approaches with exhaustive fractionation and purification is the rediscovery of compounds already used in different studies, wasting resources. In this case, both models add helpful information to suggest consistent candidates. Furthermore, another advantage of this approach relies on suggesting the molecular features as candidate(s) for structural elucidation, leveraging the MS/MS data of the crude used for the fractionation and described in the following section.

### 2.3. Identification of Neuroprotective Phytochemicals

The mass spectral features *m/z* 353.0874 and *m/z* 515.1191 were predicted as bioactive compounds associated with neuroprotective effects using the multivariate regression Elastic Net model as well as the selectivity ratio (Table 1, Figure 2). The molecular features with a selectivity ratio greater than 1 indicate that the molecular features explain 50% of the original variance and can be translated into potential neuroprotective activity. The *m/z* 303.0502 held a selectivity ratio (SR) of 2.89, *m/z* 353.0874 [M-H]- had an SR 1.78, and *m/z* 515.1191 [M-H]- had an SR 1.66 (Figure 2). By using our in-house Oregon Natural Products (ONAP) MS library containing 331 plant NPs, the *m/z* 353.0874 ion was assigned to monocaffeoylquinic acid(s) and the *m/z* 515.1191 ion to dicaffeoylquinic acid(s) [17] and their identities were verified by an LC-MS/MS comparison with authentic standards as previously described [30]. Both models successfully identified mono- and di-CQAs among the top candidates; notably, Elastic Net yielded results that highlight increased activity for di-CQAs when compared to mono-CQAs. These findings align more closely with earlier reports emphasizing the increased bioactivity of CQAs corresponding to an increased number of caffeic acid moieties [27,29,33]. Additionally, *m/z* 305.0502 was also reported as one of the constituents in *C. asiatica* corresponding to quercetin [30]. Among the highly active compounds, tentatively annotated constituents included hydroxycoumarin, myricetin 3-glucoside, caffeic acid, bisdihydroquercetin, and quercetin 7-glucuronide (Table 1). Here, we investigated the correlation of % cell viability with the concentration of di-CQAs (sum of isomers, [M-H]-, *m/z* 515.12) present in the 21 CA subfractions, obtaining a positive correlation (r = 0.613, Figure 1B). The SR and Elastic Net results do not agree completely in terms of compound importance. However, this is not unexpected, as the two analyses are quite different. Elastic Net uses a standard regression model in which the individual peaks are the features, while SR uses a regression in which the features are linear combinations of all the peaks. The data scaling is also different—as we have shown previously, we logarithmically transformed both the response and predictor variables in the Elastic Net pipeline. Finally, in the Elastic Net analysis we computed the POS and NEG mode models independently. Despite these differences, it is reassuring that the mono- and di-CQAs are identified in both approaches.

The discovery of mono- and dicaffeoylquinic acids as active compounds supports previous findings of their neuroprotective effects in both in vitro and in vivo models. For instance, evidence across various neural models such as MC65 [27,29], SH-SY5Y [34,35], and PC-12 cells [36] highlights the neuroprotective role of CQAs. Additionally, pretreatment with CQAs resulted in a significant reduction in neuronal death in rats after ischemic insult [37]. Furthermore, mono-CQAs exhibited the ability to mitigate synaptic dysfunction by either enhancing the restoration of synaptic transmission upon re-oxygenation or alleviating the aberrant alteration in hippocampal synaptic plasticity linked to memory impairment induced by exposure to β-amyloid peptides in mice models [38]. Our study further reveals that fractions A10-A12, exhibiting the highest levels of di-CQAs, correlated with enhanced cell viability. This finding corroborates our previous reports, demonstrating the protective effects of caffeoylquinic acids in *C. asiatica* against amyloid-β toxicity [27]. Furthermore, caffeoylquinic acids were found able to mitigate the cognitive deficits in the 5XFAD Alzheimer’s disease mouse model [39]. As for quercetin, its anti-inflammatory and neuroprotective effects, modulating AMPK and influencing the NF-kB and NLRP3 inflammasome pathways, have been well documented [40]. Additionally, quercetin has been implicated in promoting neuronal survival and synaptic plasticity, potentially influencing cognitive function [41,42]. While these preclinical studies provide promising insights into the neuroprotective properties of CQAs and quercetin, further well-designed clinical trials are essential to establish their efficacy and safety in the context of neurological disorders in human populations.

### 2.4. Molecular Networking for Analyzing Chemical Diversity

**Figure 3:**
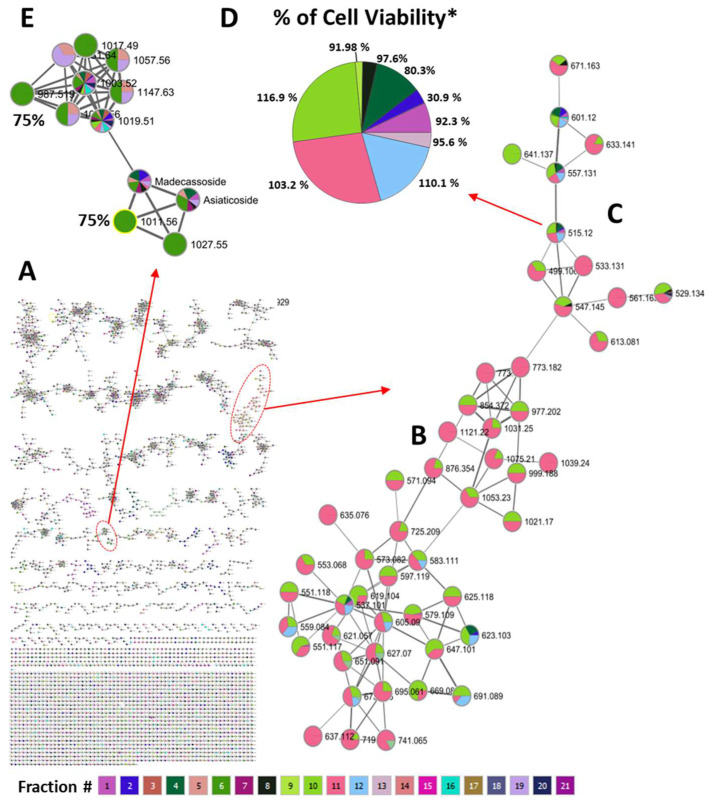
Massive molecular network for associating molecular features with sub-fraction-specific biological activities: (**A**) Generation of a massive molecular network from MS/MS data of aqueous *C. asiatica* extract. (**B**,**C**) Bioactivity mapping shows that dicaffeoylquinic acids were found in fractions providing complete protection against Aβ toxicity (panel (**D**)), whereas triterpene glycosides (panel (**E**)) were found in fractions providing partial protection (bioactivity level, 75%). GNPS constructs the molecular networks by aligning MS/MS spectra. Node assignment relates to the associated precursor ions. The edges are constructed between nodes based on cosine score, which represents the similarity of the two nodes with each other. The nodes with a cosine score of zero are completely unrelated whereas the ones that have a cosine score of 1 are identical. We chose a cut-off value of 0.70 for identifying similar nodes. Similarity matching was performed using MS/MS fragment ion information. * The percentages represent cell viability, while the size of each pie slice reflects the relative abundance of the associated molecular feature within the respective viability group. Fractions containing higher concentrations of caffeoylquinic acids exhibit favorable cell viability. Each pie slice’s color corresponds to different CAW fractions. Notably, the pies only include fractions containing the specific molecular feature indicated by its *m/z* value.

Molecular networking is a useful tool to propagate annotations of compounds sharing more than 70% of the spectral fragmentation. In addition to employing full-scan TOF-MS analysis on the 21 fractions, the crude extract containing all the components underwent an additional analysis via LC-QTOF-MS/MS to expand the characterization of the chemical diversity in the fractions. This process yielded required mass fragment (MS/MS) data for constructing a molecular network through GNPS [43]. GNPS constructs these molecular networks by aligning MS/MS spectra, where node assignment corresponds to associated precursor ions. The edges between nodes are established based on the cosine score, representing the similarity between nodes. We adopted a cut-off value of 0.70 to identify nodes with significant similarity, with the matching relying on MS/MS fragmentation information. The resultant network was visually represented using Cytoscape V3.6.1 (Figure 3). The organized MS/MS dataset comprised 5500 nodes clustered into 193 distinct groups containing three or more nodes within the network (Figure 3A). The generation of this extensive network enabled the association of molecular features with sub-fraction-specific biological activities. Our bioactivity mapping revealed the presence of dicaffeoylquinic acids in fractions that exhibited complete protection against Aβ toxicity (Figure 3B–D), contrasting with the identification of triterpene glycosides (Figure 3E) in fractions providing partial protection (bioactivity level, 75%). This integrated approach provides a powerful strategy for the rapid and resource-efficient discovery of bioactive compounds in complex plant extracts, advancing the understanding of their bioactive potential.

## 3. Materials and Methods

### 3.1. Associating Chemical Diversity of C. asiatica with % Viability from MC65 Bioassay

*Centella asiatica* water (CAW) extract was prepared as previously described [27,30]. For this study, the fractionation scheme depicted in Figure S1 was used. Initially, liquid–liquid extractions were performed, and the organic fractions were further sub-fractionated using LH-20 chromatography. CAW powder (140.5 g) was sonicated in MeOH (3 × 200 mL); MeOH insoluble materials were separated, pooled, and assigned as fraction A4 (41.3 g). The MeOH soluble fractions were pooled and dried and subjected to liquid–liquid partitioning between dichloromethane (DCM; 200 mL) and water (2 × 200 mL). The DCM layer was dried under vacuum (fraction A3; 7.8 g). The combined water layers were then partitioned with *n*-butanol (3 × 600 mL). The butanol and water layers were dried to give fractions A1 (15.9 g) and A2 (68.2 g), respectively. Sephadex LH-20 chromatography (length 40 cm, diameter 5 cm) with methanol was used to fractionate the BuOH-derived A1 and DCM-derived A3 residues, resulting in fractions A5–A13 and A14–A21, respectively. Overall, CAW constituents were distributed in 21 fractions (A1–A21; Figure S1).

### 3.2. Biological Activity in MC65 Cellular Line

MC65 cells were used because of their ability to conditionally express the C99-terminal fragment of amyloid precursor protein (APP CTF) [44]. In the absence of tetracycline, the cells are able to generate endogenous Aβ that results in cell death within 3 days. There has been evidence that links Aβ aggregates and resulting cytotoxicity with oxidative stress [45]. The maintenance of MC65 cells was performed in MEMα supplemented with 10% FBS (Gibco-BRL, Carlsbad, CA, USA) and 1 μg/mL tetracycline (Sigma-Aldrich, St. Louis, MO, USA) using the procedure described in [45,46]. Confluent cells were treated with trypsin followed by washing in PBS. The cells were resuspended in OptiMEM without phenol red (Gibco/BRL, Carlsbad, CA, USA). Cells were treated with a vehicle with or without tetracycline, or treated with fractions and without tetracycline, and then plated at 25,000 cells/well in 96-well plates. Cell viability was measured at 3 days with CellTiter 96 Aqueous Non-Radioactive Cell Proliferation Assay (Promega Corporation, Madison, WI, USA). For statistical significance and repeatability, the experiments were performed in triplicate wells for each of the CAW fractions and repeated two times.

### 3.3. Profiling of Fractions Using Flow-Injection-HRMS

Flow injection combined with high-resolution accurate mass spectrometry (HRMS) was conducted using a Shimadzu Nexera UHPLC system connected to an AB SCIEX TripleTOF^®^ 5600 (Concord, Ontario, Canada) mass spectrometer equipped with a Turbo V ionization source operated in positive and negative electrospray ion mode. For negative ion mode acquisition, the following parameter settings were used to operate the mass spectrometer: spray voltage −4200 V; source temperature 550 °C, and a period cycle time of 150 ms was used. For positive ion mode acquisitions, the instrument settings were the same as those used in the negative ion mode except that the spray voltage was set to 4500 V. The mass spectrometer was equipped with a calibrant delivery system.

For the flow-injection analysis, the flow rate was set at 0.2 mL/min utilizing aqueous methanol (20% *v/v*). A 3 µL injection volume was used. The total run time per sample was 3 min. The acquired data were aligned, deconvoluted, and normalized using Progenesis QI^TM^ V2.4 (Nonlinear Dynamics, Waters Corporation, Milford, MA, USA). This deconvolution step assembles isotopologues and adducts from the same molecular species into one molecular feature [17]. For creating a GNPS network for CAW and derived fractions, MS/MS data were acquired in data-dependent acquisition mode as previously described [30].

### 3.4. Predicting Protective Biological Activity with Mass Spectral Data

The Elastic Net analysis was conducted largely as previously described [17]. Both the response variable (bioactivity) and the predictor variable (peak intensities) were logarithmically transformed before fitting as previously described. Separate models for the POS and NEG MS modes were computed [17] and each model used an ensemble size of 1000 models. The selectivity ratio was computed using get SelectivityRatio from mdatools package v 0.11.5 [43] in R to identify discriminating *m/z* molecular features. The selectivity ratios for the molecular features were plotted using Excel.

### 3.5. Compound Identification

The molecular features identified as leads were queried by their exact mass in our in-house database as well as online databases such as the Human Metabolome Database (HMDB) (http://www.hmdb.ca/) and the METLIN database (https://metlin.scripps.edu) as previously described [30] with the following modifications: the exact mass was queried in the range of 10 ppm, and the annotations for molecular features were validated by comparing MS/MS fragments within the range of 50 ppm.

### 3.6. Molecular Networking

The MS/MS spectral data were used to create the GNPS network. MS/MS data were deposited to the GNPS repository (http://gnps.ucsd.edu). The *C. asiatica* fractions’ chemical diversity and associated % viability were used to create molecular networks using the online workflow described for Global Natural Products Social molecular networking. MSCluster was used to cluster the identical MS/MS spectra into a single spectrum. The precursor and fragment ions in the spectra were compared to the spectral libraries with mass tolerance values of ±0.01 Da for the precursor ions and ±0.05 Da for the fragment ions. The cosine score was used to compare similarities and differences of spectra with spectral libraries. The cosine score of 0.7 was used as a threshold for spectral match with libraries and the threshold for minimum matching peaks for annotating the spectral peaks was set at 6. The network was imported and visualized using Cytoscape version 3.7.

## 4. Conclusions

We have demonstrated the use of partial fractionation, HRMS, and the computational Elastic Net tool for the discovery of neuroprotective bioactives in an aqueous extract of *C. asiatica*. Mono- and dicaffeoylquinic acids, predicted as bioactive compounds by the Elastic Net method, were also in the top list of candidates resulting from the selective ratio method, underscoring the usefulness of Elastic Net as a machine learning method for bioactive component discovery. Our strategy resulted in the discovery of mono- and dicaffeoylquinic acids. Our computational approaches correctly predicted compounds previously recognized for their bioactivity using traditional approaches; dicaffeoylquinic acids have shown cognitive benefits in preclinical in vitro and in vivo models. To conclude, we report on an experimental strategy in conjunction with computational methods that streamlines the discovery and identification of bioactive constituents in botanical extracts and minimizes the need to use time-consuming traditional bioassay-guided fractionation as a primary strategy for the discovery of bioactive compounds in natural product mixtures.

## Figures and Tables

**Table 1:** Selectivity ratio and Elastic Net ranks for the experiment flow injection-TOF acquisition ion correlated with MC65 bioactivity assay.

Feature ^1^	SR ^2^	Variable Rank in Elastic Net Pipeline ^3^	Annotation ^4^	Ion Mode
1.38_303.0502 *m/z*	2.89	47 (of 119)	Quercetin	POS
1.62_257.0554 *m/z*	1.88	9 (of 85)	N/A	NEG
1.41_353.0874 *m/z*	1.78	10 (of 85)	Mono-CQAs	NEG
1.79_515.1191 *m/z*	1.66	1 (of 85)	Di-CQA’s	NEG
1.78_163.0385 *m/z*	1.58	23 (of 119)	Hydroxycoumarin	POS
1.55_461.0720 *m/z*	1.57	47 (of 85)	Myricetin 3-glucoside	NEG
1.41_179.0351 *m/z*	1.57	29 (of 85)	Caffeic Acid	NEG
1.50_539.1153 *m/z*	1.55	18 (of 119)	N/A	POS
1.41_537.1012 *m/z*	1.54	44 (of 85)	N/A	NEG
1.45_605.0894 *m/z*	1.53	45 (of 85)	N/A	NEG
1.52_513.1034 *m/z*	1.52	34 (of 85)	N/A	NEG
1.66_477.0674 *m/z*	1.52	50 (of 85)	Quercetin 7-glucuronide	NEG

^1^ Retention_*m/z*. ^2^ Selectivity ratio. ^3^ Distinct ranks were assigned to variables for each ionization mode. A selection of the 204 most prominent features was utilized to evaluate and contrast both models, with the complete set of values provided in Table S2 for positive ion mode and Table S3 for negative ion mode. ^4^ Annotated according to Phytochem Analysis 2020, 31, 722–738 [30]. MCQA—monocaffeoylquinic acid; DCQA—dicaffeoylquinic acid; POS—positive ion mode; NEG—negative ion mode; N/A—not available.

## Data Availability

Data and software are available from the authors upon request.

## Supplementary Materials

The following supporting information can be downloaded at https://www.mdpi.com/article/10.3390/molecules29040838/s1: Figure S1. Fractionation scheme. A total of 21 subfractions of CAW extract generated by solvent/solvent partitioning and LH-20 column chromatography. We analyzed each subfraction by flow-injection HRMS and correlated the features found with cytoprotective activity in an amyloid β-toxicity MC65 neuroblastoma cell model. In addition, CAW was analyzed by LC-HRMS/MS for obtaining precursor and fragment ion information for GNPS molecular network analysis. Relative polarity across fractions is indicated by “–“ and “+”.
