# Supplementary material for: Integrating High-Resolution Mass Spectral Data, Bioassays and Computational Models to Annotate Bioactives in Botanical Extracts: Case Study Analysis of C. asiatica Extract Associates Dicaffeoylquinic Acids with Protection against Amyloid-β Toxicity
Source: Molecules. 2024 Feb 13;29(4):838. doi: 10.3390/molecules29040838 (PMC10892090; doi:10.3390/molecules29040838)
Supplement: Supplementary file 1 [file molecules-29-00838-s001.zip › molecules-2827247-supplementary.pdf]

molecules

*Supplementary Materials*

# Integrating High-Resolution Mass Spectral Data, Bioassays and Computational Models to Annotate Bioactives in Botanical Extracts: Case Study Analysis of *C. asiatica* Extract Associates Dicaffeoylquinic Acids with Protection against Amyloid-β Toxicity

**Armando Alcázar Magaña [1,2,3], Ashish Vaswani [1], Kevin S. Brown [4,5], Yuan Jiang [6], Md Nure Alam [1], Maya Caruso [7], Parnian Lak [1], Paul Cheong [1], Nora E. Gray [2,7], Joseph F. Quinn [7,8], Amala Soumyanath [2,7], Jan F. Stevens [2,4,9] and Claudia S. Maier [1,2,9,*]**

[1] Department of Chemistry, Oregon State University, Corvallis, OR 97331, USA; armando.alcazarmagana@ubc.ca (A.A.M.); ashishvaswani677@gmail.com (A.V.); alammdn@oregonstate.edu (N.A.); parnian.lak@gmail.com (P.L.); cheongh@oregonstate.edu (P.C.)
[2] BENFRA Botanical Dietary Supplements Research Center, Oregon Health & Science University, Portland, OR 97239, USA; grayn@ohsu.edu (N.G.); soumyana@ohsu.edu (A.S.); fred.stevens@oregonstate.edu (J.F.S.)
[3] Life Sciences Institute, University of British Columbia, Vancouver, BC V6T 1Z4, Canada
[4] Department of Pharmaceutical Sciences, Oregon State University, Corvallis, OR 97331, USA; kevin.brown@oregonstate.edu
[5] School of Chemical, Biological, and Environmental Engineering, Oregon State University, 116 Johnson Hall, 105 SW 26th Street, Corvallis, OR 97331, USA
[6] Department of Statistics, Oregon State University, Corvallis, OR 97331, USA; yuan.jiang@oregonstate.edu
[7] Department of Neurology, Oregon Health & Science University, Portland, OR 97239, USA; maya.caruso1@gmail.com (M.C.); quinnj@ohsu.edu (J.Q.)
[8] Parkinson's Disease Research Education and Clinical Care Center, Veterans' Administration Portland Health Care System, Portland, OR 97239, USA
[9] Linus Pauling Institute, Oregon State University, Corvallis, OR 97331, USA
* Correspondence: claudia.maier@oregonstate.edu

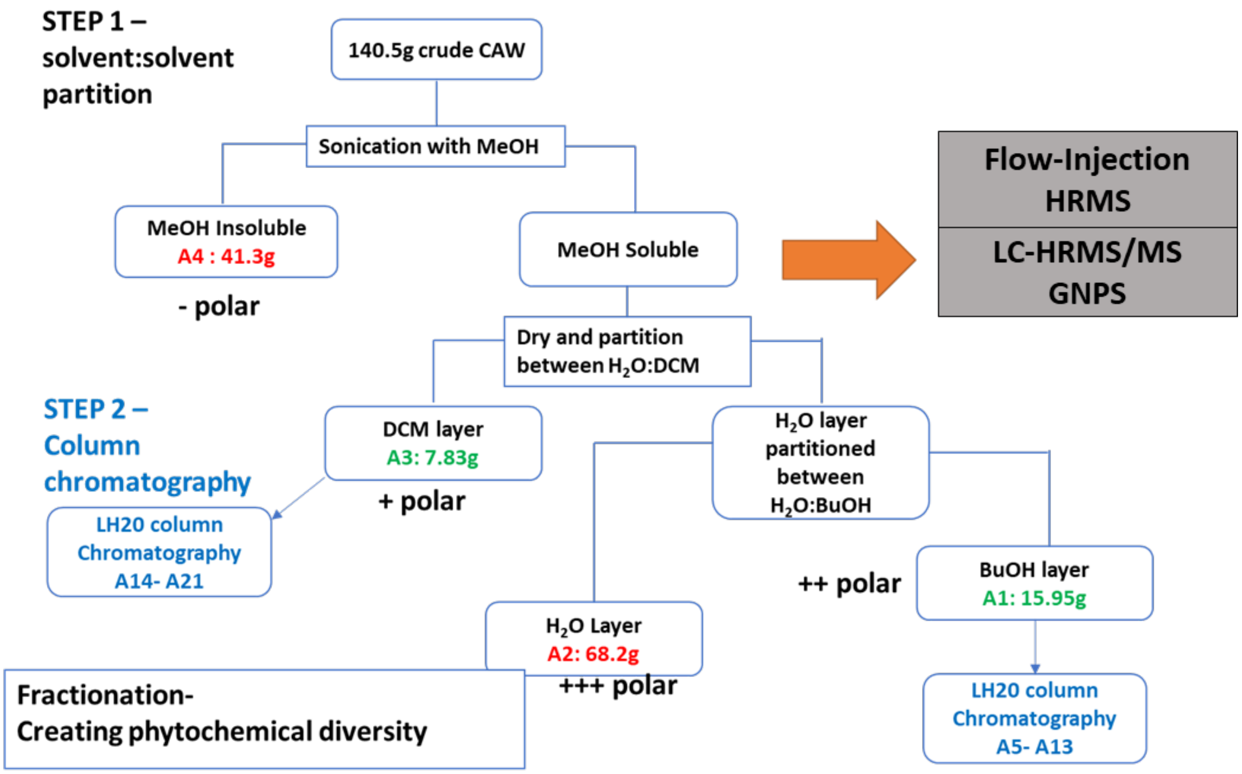


**Figure S1.** Fractionation scheme. 21 subfractions of CAW extract generated by solvent:solvent partitioning and LH-20 column chromatography. We analyzed each subfraction by flow-injection HRMS and correlated the features found with cytoprotective activity in an amyloid β-toxicity MC65 neuroblastoma cell model. In addition, CAW was analyzed by LC-HRMS/MS for obtaining precursor and fragment ion information for GNPS molecular network analysis. Relative polarity across fractions is indicated by "–" and "+".

| Molecular feature | SR |
|---|---|
| 0.69_357.0682m/z | 0.061695 |
| 1.17_568.0505m/z | 0.003033 |
| 1.22_190.0768m/z | 0.160044 |
| 1.22_191.0731m/z | 0.0862 |
| 1.22_296.6163m/z | 0.211883 |
| 1.29_238.0133m/z | 0.209372 |
| 1.30_291.0867m/z | 0.274801 |
| 1.30_305.0683m/z | 0.028729 |
| 1.30_609.1465m/z | 0.040868 |
| 1.31_285.0401m/z | 0.386458 |
| 1.31_301.0350m/z | 0.623106 |
| 1.31_579.1747m/z | 0.033141 |
| 1.32_181.0505m/z | 0.386907 |
| 1.33_235.9987m/z | 0.283181 |
| 1.34_550.1544m/z | 0.404902 |
| 1.37_611.1598m/z | 0.025595 |
| 1.37_655.1296m/z | 0.015734 |
| 1.38_181.0490m/z | 0.000769 |
| 1.38_303.0502m/z | 2.893311 |
| 1.38_369.1241m/z | 0.349537 |
| 1.41_179.0351m/z | 1.568402 |
| 1.41_353.0874m/z | 1.775024 |
| 1.41_537.1012m/z | 1.544735 |

| | |
|---|---|
| 1.42_145.0478m/z | 0.174146 |
| 1.42_266.1518m/z | 0.004587 |
| 1.42_503.1615m/z | 0.039309 |
| 1.42_707.1847m/z | 0.038408 |
| 1.43_161.0294m/z | 0.024052 |
| 1.43_233.0976m/z | 0.327369 |
| 1.43_248.1123m/z | 0.000276 |
| 1.44_177.0545m/z | 0.395364 |
| 1.44_242.0764n | 0.313693 |
| 1.44_287.0550m/z | 1.455346 |
| 1.44_380.1661m/z | 0.402307 |
| 1.44_515.1191m/z | 1.671028 |
| 1.45_301.1387m/z | 0.293446 |
| 1.45_331.1237m/z | 0.398806 |
| 1.45_381.1499m/z | 0.403051 |
| 1.45_455.1013m/z | 0.036519 |
| 1.45_549.1674m/z | 0.038088 |
| 1.45_563.1852m/z | 0.037909 |
| 1.45_571.1486m/z | 0.019339 |
| 1.45_605.0894m/z | 1.5325 |
| 1.45_666.2233n | 0.035365 |
| 1.45_685.1435m/z | 0.038043 |
| 1.45_81.0100m/z | 0.136759 |
| 1.46_1031.2511m/z | 1.181068 |
| 1.46_173.0781m/z | 0.260418 |
| 1.46_180.0630n | 0.190237 |
| 1.46_249.0946m/z | 0.300502 |
| 1.46_262.1028n | 0.31043 |
| 1.46_279.0857m/z | 0.226521 |
| 1.46_388.1213n | 0.038724 |
| 1.46_487.1666m/z | 0.036985 |
| 1.46_512.1893m/z | 1.081007 |
| 1.46_527.1568m/z | 0.034471 |
| 1.46_533.1725m/z | 0.015279 |
| 1.46_617.1554m/z | 0.038547 |
| 1.46_633.1282m/z | 0.033149 |
| 1.46_636.2187m/z | 0.03715 |
| 1.47_104.1068m/z | 0.058751 |
| 1.47_104.5399m/z | 0.00359 |
| 1.47_115.0363m/z | 0.118998 |
| 1.47_182.0791n | 0.376264 |
| 1.47_188.0657n | 0.182823 |
| 1.47_201.0498n | 0.322978 |
| 1.47_204.0604n | 0.343317 |
| 1.47_207.0708m/z | 0.144467 |
| 1.47_209.0597m/z | 0.069436 |
| 1.47_217.1048m/z | 0.035437 |
| 1.47_248.9603m/z | 0.530491 |
| 1.47_252.1114m/z | 0.387919 |
| 1.47_253.5965m/z | 0.404883 |
| 1.47_261.0943m/z | 0.360677 |
| 1.47_294.0723n | 0.310773 |
| 1.47_296.1471n | 0.312707 |
| 1.47_299.0969m/z | 0.341312 |
| 1.47_305.1209m/z | 0.354745 |
| 1.47_365.1056m/z | 0.027196 |

| | |
|---|---|
| 1.47_384.6376m/z | 0.055108 |
| 1.47_447.0925m/z | 0.843966 |
| 1.47_504.1957m/z | 0.404883 |
| 1.47_543.1311m/z | 0.032591 |
| 1.47_62.0005n | 0.005148 |
| 1.48_124.0379m/z | 0.392851 |
| 1.48_227.5722n | 0.404861 |
| 1.48_268.1009m/z | 0.423777 |
| 1.48_384.1361m/z | 0.037956 |
| 1.48_392.6290m/z | 0.03222 |
| 1.48_452.9222m/z | 0.001206 |
| 1.48_511.1626m/z | 0.023944 |
| 1.49_112.5296m/z | 0.15815 |
| 1.49_119.0699m/z | 0.358919 |
| 1.49_149.0802m/z | 0.352635 |
| 1.49_355.1236n | 0.404445 |
| 1.49_398.2411m/z | 0.859444 |
| 1.49_91.0410m/z | 0.388765 |
| 1.50_148.0375m/z | 0.353517 |
| 1.50_150.9812m/z | 0.132717 |
| 1.50_539.1153m/z | 1.545398 |
| 1.50_603.1673m/z | 0.046441 |
| 1.50_633.1415m/z | 1.226753 |
| 1.50_958.5138n | 0.008587 |
| 1.51_226.1051n | 0.370974 |
| 1.51_239.1584m/z | 0.014728 |
| 1.51_240.1208n | 0.367373 |
| 1.51_255.1084m/z | 0.382054 |
| 1.51_320.1317n | 0.292519 |
| 1.51_358.3082n | 0.116908 |
| 1.51_361.1984m/z | 0.199561 |
| 1.51_511.3385m/z | 0.009951 |
| 1.51_527.3331m/z | 0.009261 |
| 1.52_129.0538m/z | 0.29597 |
| 1.52_207.0457m/z | 0.234621 |
| 1.52_377.0844m/z | 0.031617 |
| 1.52_384.9346m/z | 0.031908 |
| 1.52_513.1034m/z | 1.524626 |
| 1.52_529.1349m/z | 1.468698 |
| 1.53_105.0196m/z | 0.382941 |
| 1.53_321.2067m/z | 0.054848 |
| 1.53_412.3207m/z | 0.008354 |
| 1.53_973.5058m/z | 0.00144 |
| 1.54_191.0565m/z | 0.348988 |
| 1.54_200.0564m/z | 0.030506 |
| 1.54_229.0667m/z | 0.146949 |
| 1.54_357.1398m/z | 0.371584 |
| 1.55_461.0720m/z | 1.570355 |
| 1.56_315.1292m/z | 0.392243 |
| 1.56_361.0745m/z | 0.017527 |
| 1.56_609.1876m/z | 0.014316 |
| 1.58_343.2123m/z | 0.009067 |
| 1.59_267.0722m/z | 0.200148 |
| 1.61_219.6155m/z | 0.038166 |
| 1.62_257.0554m/z | 1.878212 |
| 1.62_607.1265m/z | 0.010922 |

| | |
|---|---|
| 1.63_195.0644m/z | 0.383895 |
| 1.63_291.0854m/z | 0.083065 |
| 1.63_379.0827m/z | 0.028155 |
| 1.63_445.0732m/z | 0.036302 |
| 1.64_675.1287m/z | 0.016249 |
| 1.65_542.1609n | 4.68E-08 |
| 1.65_691.0992m/z | 0.00963 |
| 1.66_307.0816m/z | 0.120793 |
| 1.66_477.0674m/z | 1.522997 |
| 1.66_539.1383m/z | 0.027791 |
| 1.67_1009.4830m/z | 0.00884 |
| 1.67_215.0327m/z | 0.03779 |
| 1.67_261.0746m/z | 0.037564 |
| 1.68_255.2328m/z | 0.086941 |
| 1.68_263.0721m/z | 0.046003 |
| 1.68_277.0873m/z | 0.054633 |
| 1.68_294.1080n | 0.06986 |
| 1.68_318.2403m/z | 0.556368 |
| 1.68_377.0853m/z | 0.036924 |
| 1.68_463.0866m/z | 1.487279 |
| 1.69_310.0349m/z | 0.391028 |
| 1.70_253.2410n | 0.460207 |
| 1.70_439.0864m/z | 0.01196 |
| 1.70_701.1928m/z | 0.017073 |
| 1.71_160.0881n | 0.123625 |
| 1.71_231.0636m/z | 0.008269 |
| 1.71_276.2302m/z | 0.468145 |
| 1.71_277.1799m/z | 0.111053 |
| 1.72_167.0697m/z | 0.158417 |
| 1.72_299.1617m/z | 0.028458 |
| 1.73_313.2738m/z | 0.1441 |
| 1.73_331.2844m/z | 0.045685 |
| 1.73_352.2433m/z | 0.216793 |
| 1.73_453.3358m/z | 0.008887 |
| 1.73_479.0815m/z | 1.183939 |
| 1.74_1004.5196m/z | 0.008252 |
| 1.75_487.3412m/z | 0.008614 |
| 1.76_149.0590m/z | 0.15208 |
| 1.76_206.6398m/z | 0.101813 |
| 1.76_297.2668n | 0.476238 |
| 1.76_304.2611m/z | 0.570855 |
| 1.76_637.3045m/z | 0.006731 |
| 1.77_107.0490m/z | 0.390641 |
| 1.78_163.0385m/z | 1.584788 |
| 1.78_268.2635m/z | 0.352735 |
| 1.79_226.2158m/z | 0.283305 |
| 1.79_264.2452n | 0.464383 |
| 1.79_265.1430m/z | 0.025475 |
| 1.79_516.1262n | 1.659917 |
| 1.80_251.1638m/z | 0.126654 |
| 1.81_234.0187m/z | 0.259727 |
| 1.81_280.0241m/z | 0.39092 |
| 1.82_214.2317n | 0.385765 |
| 1.83_201.8017m/z | 0.001784 |
| 1.89_312.2534m/z | 0.073227 |
| 1.90_358.3085n | 0.324168 |

| | |
|---|---|
| 1.92_164.8363m/z | 2.95E-05 |
| 1.92_199.8047m/z | 0.00017 |
| 1.94_120.0137m/z | 0.235821 |
| 2.05_425.3776m/z | 0.002129 |
| 2.07_198.0967m/z | 0.000723 |
| 2.09_255.8221m/z | 0.000412 |
| 2.16_215.9048m/z | 0.001481 |
| 2.21_215.0695m/z | 0.1207 |
| 2.26_101.0405m/z | 0.204171 |
| 2.42_338.3419m/z | 0.18801 |

**Figure S2.** Selectivity ratio for most prominent molecular features.

| |
|---|
| 1.46_636.2187m/z,0.976143 |
| 1.48_384.1361m/z,0.971872 |
| 1.48_392.6290m/z,0.967132 |
| 1.45_563.1852m/z,0.958527 |
| 1.56_361.0745m/z,0.933042 |
| 1.38_181.0490m/z,0.932597 |
| 2.05_425.3776m/z,0.908008 |
| 1.50_603.1673m/z,0.899169 |
| 1.47_104.1068m/z,0.874875 |
| 1.44_287.0550m/z,0.857905 |
| 1.76_304.2611m/z,0.851833 |
| 2.42_338.3419m/z,0.844609 |
| 1.73_313.2738m/z,0.841983 |
| 1.79_264.2452n,0.838227 |
| 1.43_161.0294m/z,0.836286 |
| 1.37_611.1598m/z,0.836251 |
| 1.79_516.1262n,0.801490 |
| 1.50_539.1153m/z,0.784769 |
| 1.47_384.6376m/z,0.775120 |
| 1.73_331.2844m/z,0.752131 |
| 1.68_463.0866m/z,0.736052 |
| 1.51_358.3082n,0.710262 |
| 1.78_163.0385m/z,0.689532 |
| 1.82_214.2317n,0.686954 |
| 1.76_206.6398m/z,0.679012 |
| 1.78_268.2635m/z,0.676888 |
| 1.76_297.2668n,0.670230 |
| 1.71_276.2302m/z,0.657829 |
| 1.48_511.1626m/z,0.634349 |
| 1.76_637.3045m/z,0.621841 |
| 1.49_398.2411m/z,0.610729 |
| 1.46_180.0630n,0.607954 |
| 1.43_248.1123m/z,0.581380 |
| 1.70_253.2410n,0.580064 |
| 1.45_301.1387m/z,0.576685 |
| 1.48_268.1009m/z,0.556031 |
| 0.69_357.0682m/z,0.553859 |
| 1.47_104.5399m/z,0.542586 |
| 1.51_239.1584m/z,0.521342 |
| 1.90_358.3085n,0.518726 |

| |
|---|
| 1.50_958.5138n,0.508542 |
| 1.52_377.0844m/z,0.506242 |
| 1.42_266.1518m/z,0.503599 |
| 1.75_487.3412m/z,0.492380 |
| 1.76_149.0590m/z,0.471275 |
| 2.07_198.0967m/z,0.468760 |
| 1.38_303.0502m/z,0.449016 |
| 1.66_307.0816m/z,0.403072 |
| 1.73_479.0815m/z,0.378687 |
| 1.61_219.6155m/z,0.375925 |
| 1.29_238.0133m/z,0.375643 |
| 1.42_145.0478m/z,0.364537 |
| 1.53_412.3207m/z,0.363283 |
| 1.47_217.1048m/z,0.348825 |
| 2.21_215.0695m/z,0.348468 |
| 1.47_204.0604n,0.345877 |
| 1.50_633.1415m/z,0.345081 |
| 1.51_527.3331m/z,0.335108 |
| 1.73_352.2433m/z,0.330113 |
| 1.51_511.3385m/z,0.327770 |
| 1.68_318.2403m/z,0.326685 |
| 1.44_177.0545m/z,0.272564 |
| 1.79_226.2158m/z,0.262542 |
| 1.73_453.3358m/z,0.248958 |
| 1.46_512.1893m/z,0.235235 |
| 1.46_527.1568m/z,0.231113 |
| 1.44_242.0764n,0.230973 |
| 1.47_252.1114m/z,0.226425 |
| 1.72_167.0697m/z,0.211072 |
| 1.38_369.1241m/z,0.198535 |
| 1.63_195.0644m/z,0.195444 |
| 1.54_229.0667m/z,0.189209 |
| 1.81_280.0241m/z,0.187766 |
| 1.81_234.0187m/z,0.185846 |
| 1.94_120.0137m/z,0.172560 |
| 1.47_296.1471n,0.169044 |
| 1.17_568.0505m/z,0.157537 |
| 1.47_365.1056m/z,0.156546 |
| 1.47_261.0943m/z,0.143071 |
| 1.47_209.0597m/z,0.142842 |
| 1.47_294.0723n,0.142580 |
| 1.71_277.1799m/z,0.140573 |
| 1.49_355.1236n,0.135509 |
| 1.45_81.0100m/z,0.130165 |
| 1.46_173.0781m/z,0.122602 |
| 1.46_279.0857m/z,0.114921 |
| 1.72_299.1617m/z,0.114226 |
| 1.51_361.1984m/z,0.113713 |
| 1.30_291.0867m/z,0.098715 |
| 1.45_381.1499m/z,0.093743 |
| 1.47_543.1311m/z,0.090940 |

| |
|---|
| 1.79_265.1430m/z,0.088366 |
| 1.49_112.5296m/z,0.088304 |
| 1.52_129.0538m/z,0.085674 |
| 1.44_380.1661m/z,0.075481 |
| 1.48_227.5722n,0.074771 |
| 1.43_233.0976m/z,0.066033 |
| 1.46_262.1028n,0.043502 |
| 1.47_115.0363m/z,0.041941 |
| 1.77_107.0490m/z,0.037918 |
| 1.89_312.2534m/z,0.033888 |
| 1.46_249.0946m/z,0.031146 |
| 1.47_62.0005n,0.031029 |
| 1.47_305.1209m/z,0.028492 |
| 1.80_251.1638m/z,0.027538 |
| 1.71_160.0881n,0.022155 |
| 1.47_207.0708m/z,0.022004 |
| 1.47_188.0657n,0.020982 |
| 1.50_148.0375m/z,0.018821 |
| 2.26_101.0405m/z,0.017329 |
| 1.47_201.0498n,0.017057 |
| 1.49_119.0699m/z,0.015137 |
| 1.49_149.0802m/z,0.007194 |
| 1.34_550.1544m/z,0.005163 |
| 1.47_253.5965m/z,0.004648 |
| 1.47_504.1957m/z,0.004648 |
| 1.69_310.0349m/z,0.003976 |
| 1.57_675.2602m/z,0.000000 |
| 1.65_659.2864m/z,0.000000 |

**Figure S3.** List of ensemble Elastic Net importances for all POS mode peaks.

| |
|---|
| 1.44_515.1191m/z,0.993385 |
| 1.71_231.0636m/z,0.988743 |
| 1.48_452.9222m/z,0.964420 |
| 1.54_200.0564m/z,0.950291 |
| 1.52_384.9346m/z,0.923914 |
| 1.47_248.9603m/z,0.850814 |
| 1.65_691.0992m/z,0.845403 |
| 1.52_529.1349m/z,0.828675 |
| 1.62_257.0554m/z,0.778285 |
| 1.41_353.0874m/z,0.748780 |
| 1.46_487.1666m/z,0.742156 |
| 1.22_296.6163m/z,0.728621 |
| 1.42_707.1847m/z,0.719652 |
| 1.45_685.1435m/z,0.697330 |
| 1.63_445.0732m/z,0.692741 |
| 1.70_439.0864m/z,0.684225 |
| 1.64_675.1287m/z,0.672102 |
| 1.46_633.1282m/z,0.647896 |
| 1.68_377.0853m/z,0.645116 |
| 1.53_321.2067m/z,0.637866 |
| 1.31_301.0350m/z,0.627978 |

| |
|---|
| 2.09_255.8221m/z,0.625548 |
| 1.45_666.2233n,0.610582 |
| 1.30_305.0683m/z,0.600891 |
| 1.59_267.0722m/z,0.594965 |
| 1.50_150.9812m/z,0.576377 |
| 1.30_609.1465m/z,0.574029 |
| 1.53_973.5058m/z,0.547274 |
| 1.41_179.0351m/z,0.526888 |
| 1.92_164.8363m/z,0.511711 |
| 1.56_609.1876m/z,0.510920 |
| 1.32_181.0505m/z,0.492503 |
| 1.31_285.0401m/z,0.492368 |
| 1.52_513.1034m/z,0.490138 |
| 1.83_201.8017m/z,0.486536 |
| 1.67_215.0327m/z,0.485084 |
| 1.92_199.8047m/z,0.482812 |
| 1.74_1004.5196m/z,0.475229 |
| 1.70_701.1928m/z,0.450704 |
| 1.37_655.1296m/z,0.446044 |
| 1.45_549.1674m/z,0.437753 |
| 1.58_343.2123m/z,0.434060 |
| 1.22_190.0768m/z,0.431715 |
| 1.41_537.1012m/z,0.431659 |
| 1.45_605.0894m/z,0.421140 |
| 1.68_294.1080n,0.391232 |
| 1.55_461.0720m/z,0.387356 |
| 1.31_579.1747m/z,0.379605 |
| 1.68_263.0721m/z,0.348223 |
| 1.66_477.0674m/z,0.346170 |
| 1.22_191.0731m/z,0.340109 |
| 1.68_277.0873m/z,0.330220 |
| 1.68_255.2328m/z,0.303393 |
| 1.42_503.1615m/z,0.300544 |
| 1.56_315.1292m/z,0.298810 |
| 1.67_1009.4830m/z,0.278626 |
| 1.47_447.0925m/z,0.273950 |
| 1.62_607.1265m/z,0.268125 |
| 1.66_539.1383m/z,0.266531 |
| 1.46_388.1213n,0.260211 |
| 1.51_320.1317n,0.256745 |
| 1.46_617.1554m/z,0.252164 |
| 1.63_379.0827m/z,0.242498 |
| 1.65_542.1609n,0.237667 |
| 1.54_191.0565m/z,0.235819 |
| 1.47_299.0969m/z,0.233165 |
| 1.45_455.1013m/z,0.226382 |
| 1.46_1031.2511m/z,0.222524 |
| 1.33_235.9987m/z,0.202234 |
| 2.16_215.9048m/z,0.172928 |
| 1.46_533.1725m/z,0.169044 |
| 1.51_240.1208n,0.156574 |

| |
|---|
| 1.49_91.0410m/z,0.125754 |
| 1.45_571.1486m/z,0.121358 |
| 1.67_261.0746m/z,0.069195 |
| 1.45_331.1237m/z,0.060759 |
| 1.47_182.0791n,0.058338 |
| 1.51_226.1051n,0.047560 |
| 1.54_357.1398m/z,0.047384 |
| 1.53_105.0196m/z,0.039315 |
| 1.51_255.1084m/z,0.038672 |
| 1.52_207.0457m/z,0.033886 |
| 1.48_124.0379m/z,0.023481 |
| 1.63_291.0854m/z,0.009875 |
| 1.45_869.2179m/z,0.000000 |

**Figure S4.** List of ensemble Elastic Net importances for all NEG mode peaks.
